# A Leukocyte Migration Assay Assists Understanding of Interleukin-1β-Induced Leukocyte Migration Into Preterm Mouse Uterus

**DOI:** 10.3389/fphar.2022.898008

**Published:** 2022-05-25

**Authors:** Han Lee, Vaishvi Patel, Meghan Onushko, Xin Fang, Sylvain Chemtob, David Olson

**Affiliations:** ^1^ Olson Laboratory, Department of Physiology, University of Alberta, Edmonton, AB, Canada; ^2^ Olson Laboratory, Faculty of Science, University of Alberta, Edmonton, AB, Canada; ^3^ Olson Laboratory, Department of Obstetrics/Gynecology, University of Alberta, Edmonton, AB, Canada; ^4^ Chemtob Laboratory, Departments of Pediatrics and Ophthalmology/Pharmacology, University of Montreal, Montreal, QC, Canada; ^5^ Olson Laboratory, Department of Pediatrics, University of Alberta, Edmonton, AB, Canada

**Keywords:** rytvela, preterm birth, leukocyte migration assay, chemotaxis, inflammation, neutrophils, mouse, fetal

## Abstract

Neutrophils and other leukocytes invade the mouse uterus at term birth, which is normal for activating the uterus for labor. To better understand the regulation of this migration at term and interleukin (IL)-1β—induced preterm birth, we developed a mouse leukocyte migration assay (mLMA) and used it with rytvela, an IL-1 receptor allosteric antagonist. The mLMA uses term peripheral blood leukocytes that migrate in a Boyden chamber in response to a chemoattractant. We tested several mouse uterine tissues after homogenization and sedimentation of debris for chemoattractant activity. The most active chemoattractant homogenate came from the mouse lower uterus on gestational day (GD) 18.5. Using flow cytometry, we demonstrated that 99% of the cells that migrate are neutrophils. IL-1β administered on GD 16 stimulated neutrophil migration and invasion into the uterus and the fetal brain along with preterm birth on GD 17. Preterm birth and the increased leukocyte invasion of the maternal uterus and fetal brain were all blocked by the co-administration of rytvela. To test where the site of IL-1β action might be, we examined the potency of lower uterine chemoattractant and the activation of leukocytes following IL-1β +/- rytvela administration. IL-1β did not increase lower uterus homogenate chemoattractant activity, but it significantly (*p* < 0.05) increased leukocyte activation as defined by cytokine and chemokine expression. Rytvela blocked this activation of leukocytes by IL-1β. We conclude that IL-1β stimulates preterm birth in mice by increasing leukocyte activation leading to increased uterine and fetal brain leukocyte invasion.

## 1 Introduction

Invasion of circulating leukocytes into gestational tissues is a conserved phenomenon of term and preterm birth (PTB) among mammalian species. In humans, leukocyte densities in the fetal membranes, decidua, cervix, and myometrium increase significantly at term labor (TL) (Thomson et al., 1999; Osman et al., 2003), but a greater proportion of total innate lymphoid cells infiltrate the decidua parietalis in preterm labor (PTL) (Xu et al., 2018). Moreover, elevated leukocyte counts are found in the amniotic fluid of women who delivered preterm and exhibited an infectious etiology (Romero et al., 1991). In mice, both full- and sub-PTB-inducing doses of the Gram-negative bacterial mimic lipopolysaccharide (LPS) stimulate neutrophil infiltration of fetal membranes, and a PTB-inducing dose of LPS induces infiltration of the fetal lung and kidneys (Hudalla et al., 2018). Once leukocytes invade, they release an array of matrix metalloproteases, prostaglandins, cytokines, chemokines, and other effectors that amplify the inflammatory event, activate the uterus for labor (e.g., altered expression of uterine activation proteins), remodel the cervical extracellular matrix, and break down the fetal membranes (Orsi and Tribe, 2008; Leimert et al., 2019a; Leimert et al., 2019b). These events cause parturition.

We and others have studied the mechanisms of leukocyte migration at term and preterm in pregnant women, rats, and guinea pigs (Gomez-Lopez et al., 2009; Yuan et al., 2009; Gomez-Lopez et al., 2013; Gomez-Lopez et al., 2015; Takeda et al., 2017). Homogenates from human fetal membranes, amnion and chorion, release a chemoattractant whose levels rise as labor approaches. The responsiveness of the leukocytes to the chemoattractant also increases as labor approaches. We developed a leukocyte migration assay (LMA) to study this phenomenon in rats, guinea pigs, and humans using a Boyden chamber that mimics some of the early *in vivo* actions of leukocyte invasion (Gomez-Lopez et al., 2013; Gomez-Lopez et al., 2015; Takeda et al., 2017).

Other work by our team demonstrated that PTB and arrested fetal organogenesis are induced in mice by administration of IL-1β (Nadeau-Vallee et al., 2015; Nadeau-Vallee et al., 2017), a model for sterile inflammation (Romero et al., 2014), or by lipoteichoic acid (LTA), a Gram-positive bacterial mimic, and LPS. LTA and LPS interact with the toll-like receptors (TLR) 2 and 4, respectively, which stimulate IL-1β production that leads to PTB. This was made evident when PTB was blocked by the administration of rytvela, a 7-amino acid allosteric antagonist of the IL-1 receptor (IL-1R). Together, these reports suggest that IL-1β is a central mediator of PTB, regardless of whether an infection is present or not.

The purpose of this study was to develop a mouse leukocyte migration assay (mLMA) and then use it along with rytvela to interrogate the mechanism of IL-1β-induced leukocyte migration in the mouse at PTB. We hypothesized that IL-1β stimulates both leukocyte invasion and migration into the maternal uterus and fetal brain during PTB, and it does so by increasing both the chemoattractant activity in the uterine homogenate and leukocyte activation.

## 2 Materials and Methods

### 2.1 Study Approval

Animal studies at the University of Alberta were conducted following the Canadian Council on Animal Care Guidelines and Policies with approval from the University of Alberta’s Animal Care and Use Committee (Biosciences, Health Sciences or Livestock) (Protocol No. AUP00003510). Human leukocytes were obtained following written, informed consent; the University of Alberta/Alberta Health Services Institutional Review Board approved the study (Protocol No. Pro00069209).

### 2.2 Collection of Human Blood

Human whole blood was collected from healthy, pregnant women in TL at the Royal Alexandra Hospital (Edmonton, AB, Canada) after written informed consent. Labor was defined as a cervical dilation of greater or equal to 4 cm in the presence of uterine contractions. Women with a clinical infection, premature rupture of membranes, diabetes mellitus, immunological problems, non-singleton pregnancies, intrauterine growth restriction, preeclampsia or dysfunctional labor, and recipients of progesterone or oxytocin were excluded from the study.

### 2.3 Preparation of Mouse Chemoattractant Homogenates

Groups of mice were euthanized on GD15, 17, 18, 18.5, 19 during spontaneous labor and post-partum (PP)-1. Mouse upper or lower uterus, placenta, fetal membranes, or cervix were collected, homogenized in PBS, and centrifuged to pellet cell debris. The supernatant was collected and frozen. It was later thawed and tested for chemoattractant activity using the LMA.

### 2.4 Leukocyte Migration Assay

Total leukocytes were isolated from whole blood (collected from either humans or mice, Section 2.3) using HetaSep (Stemcell Technologies Canada Inc., BC, Canada) and following the manufacturer’s protocols. Leukocytes were then resuspended in Roswell Park Memorial Institute medium 1,640 (RPMI 1640) and diluted to 200,000 cells per 100 μl. Chemoattractants (50 μl) were loaded into the lower wells of a 96-well chemotaxis chamber (Neuro Probe Inc., MD, United States), and a polycarbonate filter (3 µm pores) was placed on top. Total leukocytes were loaded into the upper wells and were left to migrate towards the chemoattractant at 37°C in 5% CO_2_ for 30 min. Chemoattracted leukocytes were then transferred to a 96-well, black, clear-bottom plate. Measurements were taken in triplicate determination. For quantitation, Hoechst 33342 (1pM) (Thermo Fisher Scientific) was added to reach a final volume of 100 µl per well, and fluorescence was measured using a Fluoroskan Ascent microplate reader (Thermo Fisher Scientific). The number of chemoattracted leukocytes was calculated using 4 parameter logistic regression.

### 2.5 Inducing PTL in Mice Using IL-1β

CD-1 mice were ordered from Charles-River Laboratories Canada (Montreal, QC, Canada) and housed by Health Sciences Laboratory Animal Services at the University of Alberta (Edmonton, AB, Canada) on a standard diet. The mice were anesthetized under isoflurane, and a 1.5 cm-tall median incision was made in the lower abdominal wall. The lower segment of the right uterine horn was exposed, IL-1β (3 µg) or vehicle (0.9% saline) was injected between two fetal membranes, and the abdominal muscle layer and skin were sutured. Each treatment group was further divided into two groups where either Rytvela (1 mg/kg, Elim Biopharmaceuticals, Hayward, CA) or vehicle (0.9% saline) was injected intraperitoneally in a total volume of 100 µl saline. The first dose was given 30 min prior to administration of stimuli and three doses every 12 h thereafter. In summary, there were four treatment groups of vehicle alone (sham); IL-1β and vehicle; IL-1β, rytvela, and vehicle; and rytvela and vehicle (n = 5). Animals were monitored for the onset of PTL using infrared video camera recording commencing at GD16.5. Mice were euthanized at the onset of PTL or at GD 17. Tissues were collected (lower uteri, upper uteri, placentas, fetal membranes, and cervices) and normalized to wet tissue weight (100 mg/ml) in Dulbecco’s Modified Eagle’s Medium (DMEM) F-12 (Thermo Fisher Scientific, MA, United States), homogenized by TissueLyser II (QIAGEN, Hilden, Germany), and centrifuged at 4°C, 13,000 × *g* for 10 min to extract total protein. Maternal whole blood (1–2 ml) was collected *via* cardiac puncture. Whole fetuses (n = 2 per dam) were also collected.

### 2.6 Immunohistochemistry

Six cryosections (7 µm) per treatment group were prepared from snap-frozen lower uteri and fetal heads (n = 2 per dam) and stained for neutrophils using a goat anti-mouse Ly-6G antibody (Invitrogen, Carlsbad, CA, United States) and a rat anti-goat secondary antibody tagged with an Alexa Fluor 488 fluorescence marker (Invitrogen). Cells were counterstained using Hoechst 33,342, and tissue homology was confirmed with a hematoxylin and eosin stain (Thermo Fisher Scientific). Four different fields (×20 optical zoom) were counted per section by two observers blinded to the specimen details. Areas containing blood vessels and leukocytes within blood vessels were excluded. The arithmetic mean was calculated for each sample between the two observers.

### 2.7 RNA Extraction and RT-qPCR

Total RNA was extracted from mouse whole blood using the RNeasy protect animal blood system (Qiagen, Hilden, Germany). RNA concentrations were determined using a NanoDrop 1,000 spectrophotometer to measure the optical density (OD) photometrically at 280 and 260 nm cDNA was synthesized from 500 ng RNA using qScript cDNA SuperMix (Quanta Biosciences, MA, United States). Primers for mouse *Il-1β*, *Il-6*, *Tnf-α* (tumor necrosis factor α), and *Ccl2* (C-C motif chemokine ligand) were designed using the National Center for Biotechnology Information’s Primer Blast (Table 1). Quantitative gene expression analysis was performed on an *i*Cycler IQ (Bio-Rad, CA, United States) using SYBR Green Master Mix (Bio-Rad). Target gene levels were expressed relative to *CypA*.

**TABLE 1:** The RT-qPCR primers for mouse cytokines.

	Forward primer	Reverse primer
IL-1β	5′-AGA​TGA​AGG​GCT​GCT​TCC​AAA-3′	5′-GGA​AGG​TCC​ACG​GGA​AAG​AC-3′
IL-6	5′-CAA​CGA​TGA​TGC​ACT​TGC​AGA-3′	5′-TCT​CTC​TGA​AGG​ACT​CTG​GCT-3′
TNF-α	5′-GCC​TCT​TCT​CAT​TCC​TGC​TTG-3′	5′-CTG​ATG​AGA​GGG​AGG​CCA​TT-3′
CCL2	5′-GCT​CAG​CCA​GAT​GCA​GTT​A-3′	5′-TGT​CTG​GAC​CCA​TTC​CTT​CT-3′

### 2.8 Statistics

The data were tested by the D'Agostino-Pearson normality test using GraphPad Prism 8.0. All data were normally distributed. Statistical significance was tested by one-way ANOVA. When a significant F value was found, means were differentiated using Tukey’s *post hoc* multiple comparisons test. Significance was achieved at *p* ≤ 0.05.

## 3 Results

### 3.1 Development of a Mouse Leukocyte Migration Assay

**FIGURE 1:**
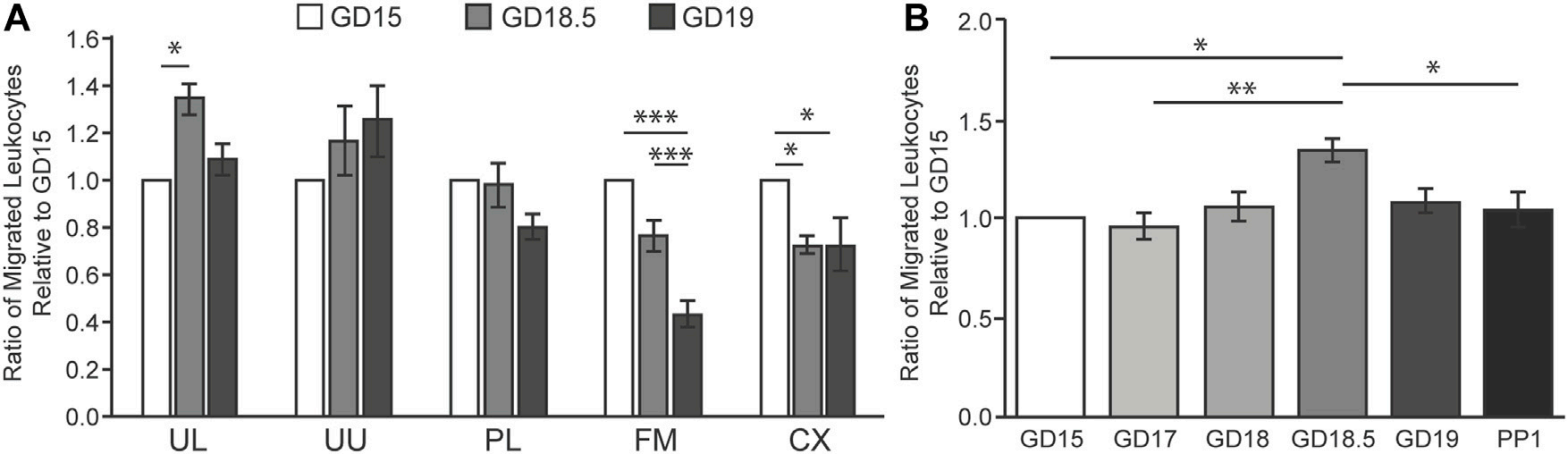
Intrauterine tissue chemotactic factor in mice during late gestation using human TL leukocytes in LMA. **(A)** Total protein was extracted from mouse intrauterine tissues at GD 15, 18.5, or 19 and pooled (n = 6 per GD). Abbreviations: UL, uterus lower UU, uterus upper; PL, placenta; FM; fetal membranes; CX, cervix. **(B)** Total protein was extracted from the lower uterus from mice at GD 15, 17, 18, 18.5, 19, or 1 day postpartum (PP1) and pooled (n = 6 per GD). Relative migration of human leukocytes (n = 6) was expressed as the averaged ratio of migrated leukocytes towards each chemoattractant compared to GD 15 for each trial. *p < 0.05, **p < 0.01, ***p < 0.001.

First, we identified the best source and timing of the chemoattractant using TL human leukocytes. We compared the chemotactic activities of homogenates obtained from the lower and upper uterus, placenta, fetal membranes, and cervix on GDs 15, 18.5, and 19 (Figure 1A). The lower uterus produced the most chemoattractant activity, which increased from GD 15 to 18.5. This increase preceded delivery by about 0.5 days which correlated with a role in initiating labor. Both the source of the chemoattractant activity and the timing of its increase relative to delivery are similar to our rat study (Gomez-Lopez et al., 2013). We then compared extracts of homogenized lower uterine tissue with greater clarity from 4 days before to 1 day after delivery using TL human leukocytes (Figure 1B). Again, we found a statistically significant increase (*p* < 0.05) in chemoattractant potency at GD 18.5, approximately 12 h before delivery. Chemoattractant potency decreased in the lower uterus at 1 day postpartum, coinciding with expulsion of the products of conception.

### 3.2 mLMA With Mouse TL Leukocytes

**FIGURE 2:**
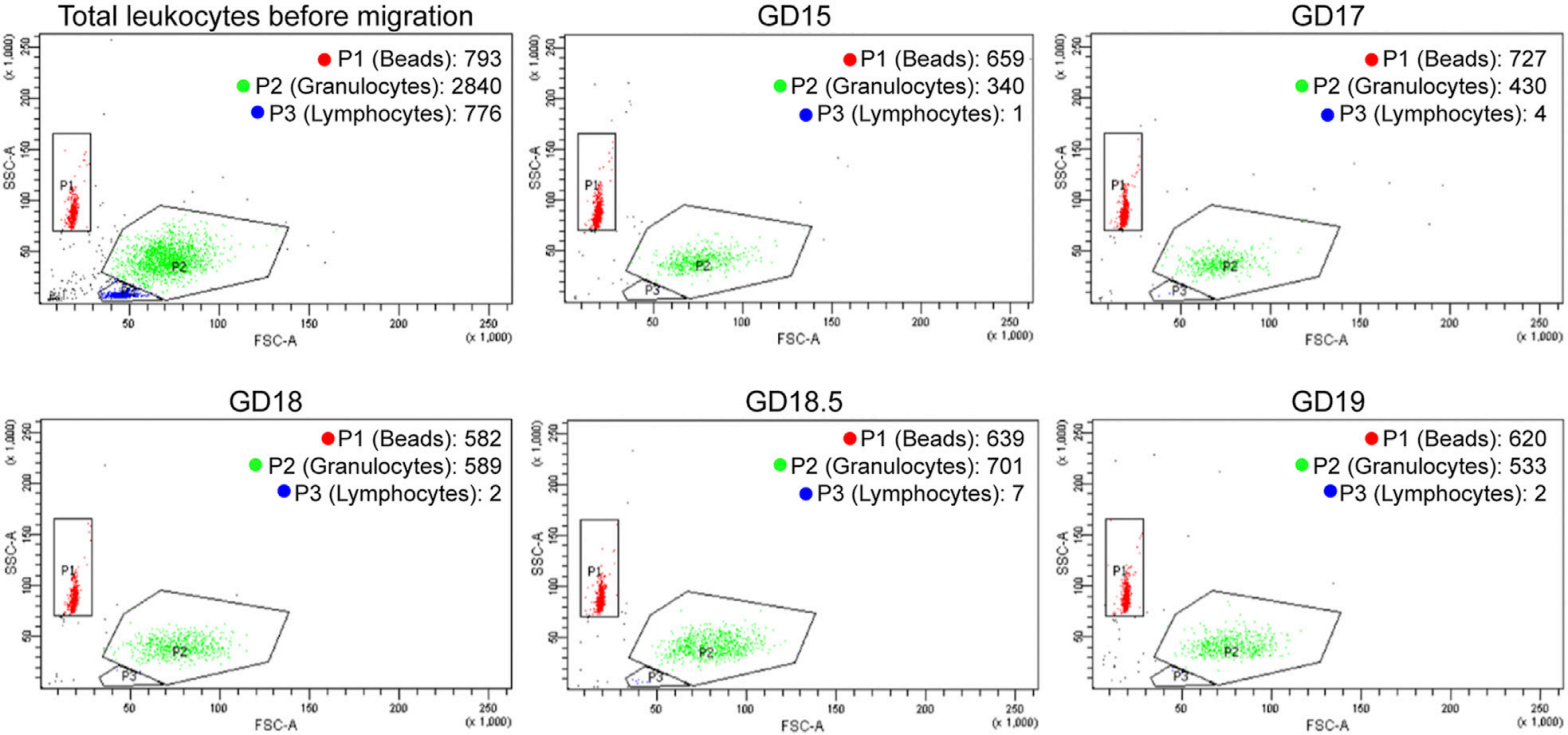
Representative FACS distribution of mouse leukocytes in LMA using mouse lower uterine chemoattractant during late gestation. The upper left diagram (Total Leukocytes) describes the distribution of GD 15 mouse leukocytes before migration (granulocytes and lymphocytes) plus beads. The other diagrams describe the distribution of leukocytes plus beads that migrate in the LMA during late gestation. The number of granulocytes migrated increases until GD 18.5 and then decreases on GD 19. There is no migration of lymphocytes.

Once the ideal source and timing of chemoattractant were determined, the next step was to test the relative attraction of pregnant term mouse leukocytes to mouse chemoattractant (from GD 15–19) using flow cytometry (Figure 2). We followed the migration of granulocytes and lymphocytes; the beads demonstrated the flow through the chamber. The flow of control beads was consistent through this 4-day period, but there was no attraction of lymphocytes. Granulocytes (neutrophils) comprised 99% of the migrated cells. A marked increase in granulocyte numbers occurred on GD 18.5 and fell on GD 19. These data indicate that granulocytes and homogenate chemoattractant reach their greatest migration attraction on GD 18.5. The implications are that the chemoattractant influences granulocyte activation and migration, and they activate the uterus for delivery.

### 3.3 IL-1β Induces Neutrophil Invasion of Maternal Uterus and Fetal Brain, and PTB, Resulting in Fetal Death*,* but Rytvela Blocks These Actions

**FIGURE 3:**
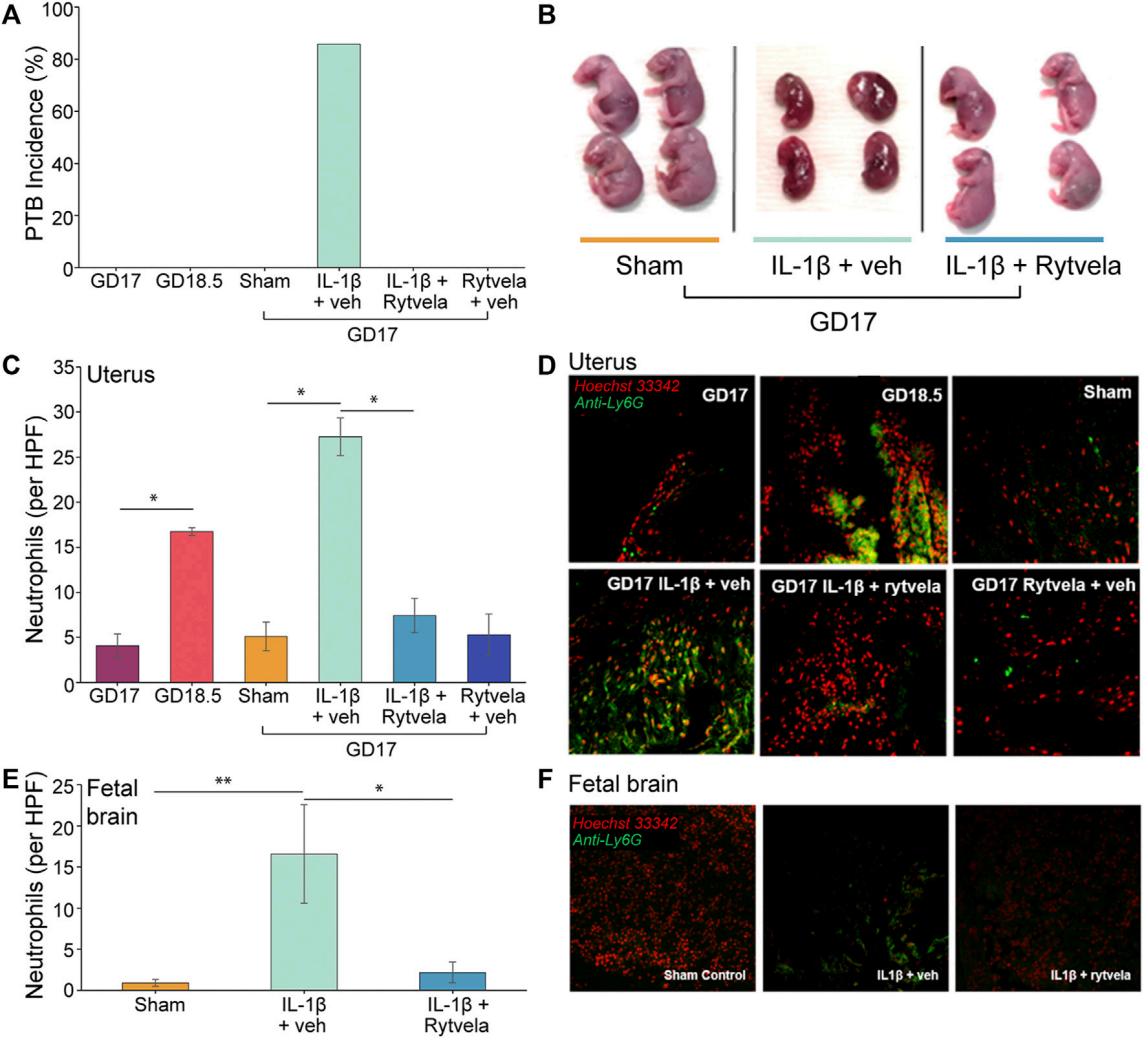
Effect of IL-1β and rytvela on preterm delivery and neutrophil invasion of mouse uterus and fetal brain. **(A)** PTB occurs only in IL-1β-stimulated mice. This is inhibited by co-administration of rytvela (n = 5 per group). **(B)** Representative images of GD 17 fetuses. Those dams treated 24 h earlier with IL-1β alone (centre) were undergoing resorption whereas dams co-treated with rytvela had normal GD 17 fetuses. **(C)** Counts of neutrophils per high powered field (20x optical zoom) in mouse lower uterus (n = 5). **(D)** Representative histological images of the mouse lower uterus stained for DNA (Hoechst 33342, red stain) and a neutrophil surface marker (anti-Ly6g, green stain). **(E)** Counts of neutrophils per high powered field (20x optical zoom) in mouse fetal brains (n = 3 dams per group, n = 2 fetuses per dam). **(F)** Representative histological images of the mouse fetal brain stained for DNA (Hoechst 33,342, red stain) and a neutrophil surface marker (anti-Ly6g, green stain). *p < 0.05, **p < 0.01.

Given the central role that IL-1β plays in mediating both sterile and infectious etiologies of PTB, IL-1β was chosen to induce PTB in our mouse model to study the corresponding neutrophil invasion of the maternal uterus and fetal brain (Figure 3). This is the first investigation of neutrophil invasion in these tissues, as other studies did not examine this question (Romero et al., 2014; Nadeau-Vallee et al., 2015; Nadeau-Vallee et al., 2017). Compared to sham controls, IL-1β administered on GD 16 resulted in high rates of PTB (86%) on GD 17 (Figure 3A), increased fetal death (Figure 3B), and high levels of Ly-6G + neutrophils in the lower uterus and fetal brain (Figures 3C–E). Similar levels of Ly-6G+ were detected on GD 18.5 in the uteri of untreated mice (*p* < 0.05 compared to GD 17 control) (Figures 3C,D). Rytvela co-administration in mice stopped PTB, prevented fetal death, and blocked the increase in neutrophil density in the maternal uterus and fetal brain (*p* < 0.05, Figures 3A–E). Next, we explored whether IL-1β increases leukocyte migration in addition to stimulating preterm delivery.

### 3.4 IL-1β Stimulates Pregnant Mouse Leukocyte Migration

**FIGURE 4:**
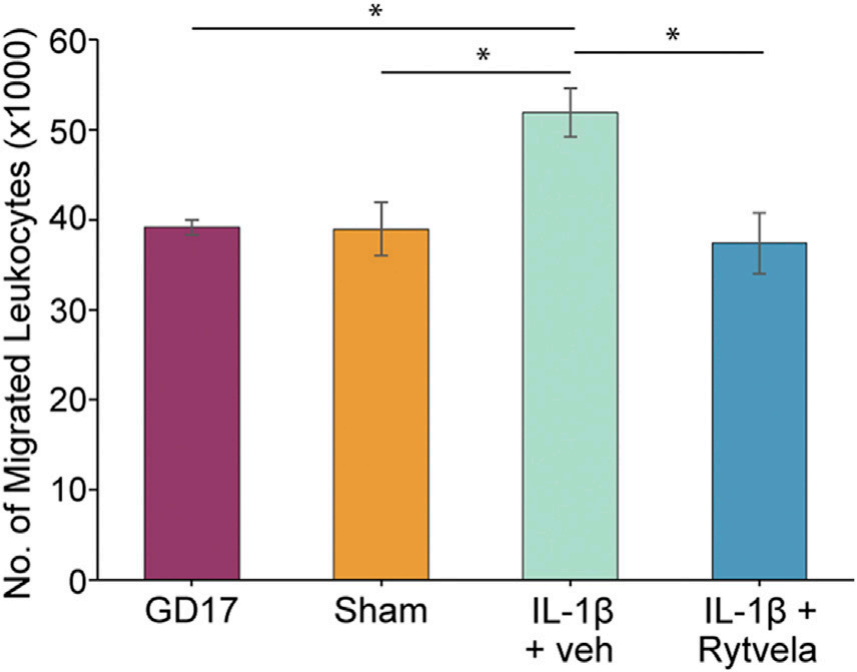
Effect of IL-1β without or with rytvela on GD 16 on migration of mouse leukocytes from pregnant mice at GD 17 using mouse chemoattractant from GD 18.5 lower uterus. Interleukin-1β administration on GD 16 significantly increased leukocyte migration on GD 17. Rytvela co-administration blocked this increase in leukocyte migration (n = 3–4). *p < 0.05.

We observed that the administration of IL-1β on GD 16 that produces PTB on GD 17 also increased leukocyte migration on GD 17 (*p* < 0.05). Co-administration of rytvela blocked this IL-1β-induced migration (Figure 4). The next question was, where does the action of IL-1β lie–at the level of the uterine chemoattractant or at the level of leukocyte activation?

### 3.5 Testing the Effects of IL-1β Administration on Homogenate Chemotactic Activity and Leukocyte Activation

**FIGURE 5:**
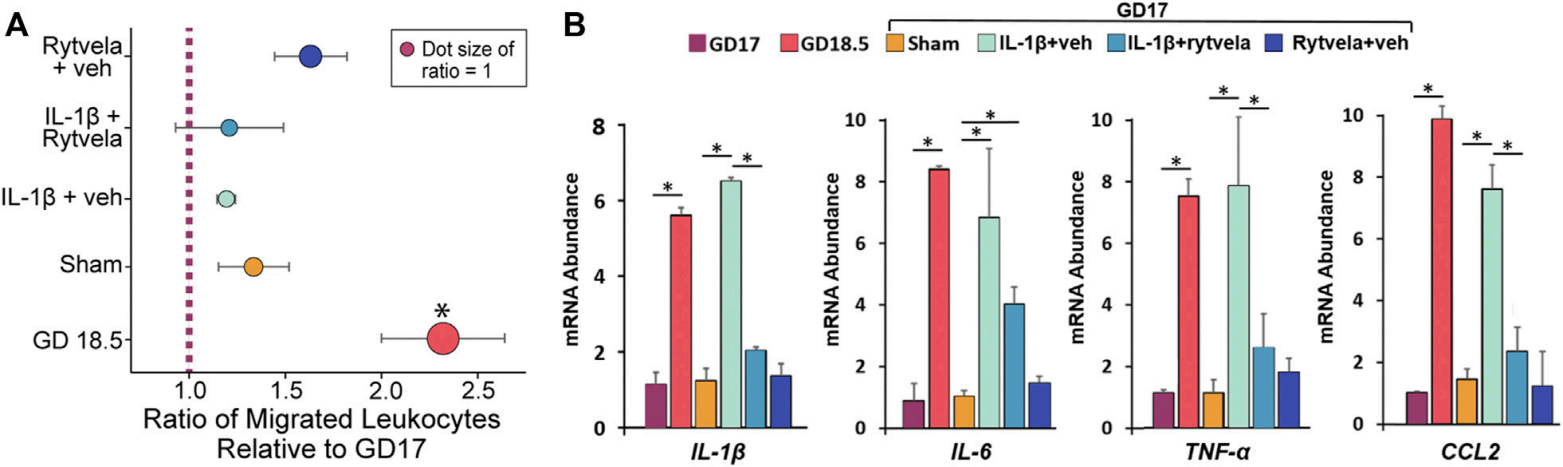
Effect of IL-1β administration without and with rytvela on chemoattractant (A) and cytokine mRNA abundance in peripheral leukocytes in pregnant mice (B). **(A)** The relative migration of leukocytes was expressed as the ratio of migrated leukocytes towards chemoattractant compared to GD17 or sham for each trial, then averaged. **(B)** The term increase and effect of IL-1β alone or with rytvela on leukocyte cytokine qPCR values are shown on GD 17 and 18.5 in untreated dams and on GD 17 in dams treated with vehicle, IL-1β alone or with rytvela and rytvela plus vehicle. N = 5–7, *p < 0.05. In **(A)**, * denotes significance relative to ratio of 1.

We tested whether systemically administered IL-1β on GD 16 stimulated homogenate chemotactic activity and leukocyte activity (Figure 5). There was a naturally occurring increase in chemoattractant activity on GD 18.5 compared to GD 17. However, administration of IL-1β on GD 16 did not stimulate a similar increase in chemoattractant activity on GD 17 (Figure 5A).

The mRNA abundance of IL-1β, IL-6, TNF-α, and CCL2 in peripheral mouse maternal leukocytes increased naturally on GD 18.5 compared to GD 17 (Figure 5B, *p* < 0.05). Administration of IL-1β on GD 16 stimulated a similar increase in mRNA expression of these cytokines on GD 17 (*p* < 0.05), and this was blocked by co-administration of rytvela (*p* < 0.05). These data suggest that the action of IL-1β is at leukocyte activation and not lower uterus chemoattractant activity.

## 4 Discussion

We established and optimized the mLMA using lower uterine extract chemoattractant in this study. The highest level of extract chemotactic activity occurred on GD 18.5 about 12 h prior to normal delivery (Figures 1, 2). This assay was then used to examine the mechanism of IL-1β-stimulated neutrophil invasion of the pregnant mouse uterus at PTB, an action that was blocked by co-treatment with rytvela. Indeed, administration of IL-1β on GD 16 stimulated both leukocyte invasion and migration into the maternal uterus and fetal brain at PTB as hypothesized (Figure 3). However, the data demonstrate that our hypothesis on the actions of IL-1β was only partially correct. Systemically-administered IL-1β stimulated leukocyte migration and leukocyte activation (Figure 4) but not an increase in lower uterine homogenate chemoattractant activity (Figure 5A). These results are similar to data we have from pregnant women (not shown), where IL-1β did not increase the chemoattractant activity of fetal membranes (the highest source of activity), but it did stimulate peripheral leukocyte activation and migration.

The chemoattractants arise from extracts of the uterus, fetal membranes, and placenta. In mice, we found that the highest level of activity that increased at term was from the lower half of the uterus. This result coincides with an earlier study in rats (Gomez-Lopez et al., 2013). In guinea pigs and humans, in contrast, the highest activity that increased at term was found in the fetal membranes (amniochorion) (Osman et al., 2003; Gomez-Lopez et al., 2009; Gomez-Lopez et al., 2013; Gomez-Lopez et al., 2015). Extracts from other uterine tissues, e.g., placenta and cervix, exhibit chemotactic activity, but they do not increase at term, and some even decrease (Figure 1A). It is likely that the increase in lower uterus or fetal membrane extract chemotactic activity at term is a signal for the initiation of the birth process. The similarity of increasing activities in late gestation from the lower uterus in the polytocous rodent (mouse and rat) species, a maternal tissue, versus the amniochorion in the monotocous primate, human, which are fetal tissues, indicates where the control for the timing of birth may lie. While guinea pigs are considered polytocous since the number of fetuses ranges from 1 to 8 (2–4 is the norm), they are not rodents (D'Erchia et al., 1996), and their placental anatomy and lack of a progesterone withdrawal at the end of pregnancy are much closer to that of humans (Mitchell and Taggart, 2009). The source of chemoattractive extract from the guinea pig fetal membranes is another commonality it shares with human parturition physiology (Mitchell and Taggart, 2009).

This study also showed that TL human leukocytes migrate in response to chemoattractants from mouse uterine tissue extracts (Figure 1). We also have unpublished data that the reverse occurs, i.e., chemoattractants from human fetal membrane extracts attract term mouse leukocytes. These data suggest that the chemotactic components of the extracts from the human fetal membrane and mouse lower uterus are similar. The receptors on leukocytes (primarily neutrophils) for these chemoattractant components are also likely conserved between species. Altogether, the data suggest that different mammalian species use similar chemoattractants and similar responsive elements in the leukocytes.

The uniformity that occurs in mouse pregnancy and parturition allows for precise timing of its component events. In this study, we demonstrated that the highest level of chemotactic activity in the lower uterus extracts was at 18.5 days or 12 h before normal delivery. This timing suggests that the leukocyte invasion of the uterine tissue is a necessary early event in the initiation of parturition. This notion was confirmed when we stimulated preterm delivery by IL-1β administration and observed uterine invasion of leukocytes. Rytvela co-administration blocked the neutrophil invasion of the uterus and also preterm delivery.

The role of IL-1β at preterm in the mouse is clear; it stimulates leukocyte activation in terms of cytokine and chemokine release (Figure 5B) (Nadeau-Vallee et al., 2015), neutrophil invasion of the uterus and fetal brain (Figure 3) (Nadeau-Vallee et al., 2017), and increased migration in general (Figure 4). These data support part of our hypothesis. However, IL-1β administration does not cause an increase in the potency of the lower uterine chemoattractant (Figure 5A). Our data contribute to the existing work that draws an association between PTB and leukocyte invasion (Romero et al., 1991; Shynlova et al., 2013a; Hudalla et al., 2018; Xu et al., 2018). We propose that IL-1β enhances the ability of circulating leukocytes to migrate and invade the uterus. Future studies will examine the profile of chemokine receptors expressed on these mouse leukocytes to assess whether it is similar to those expressed on GD 18.5.

Leukocytes, especially granulocytes plus monocytes and macrophages, are activated in late gestation to invade the uterus. There they release their inflammatory products to further the transition of the uterus from a pregnant to a parturient organ (Robertson et al., 1994; Orsi and Tribe, 2008; Gomez-Lopez et al., 2010; Gomez-Lopez et al., 2014). Shynlova *et al.* observed a significant increase in neutrophil and macrophage numbers in the mouse decidua on GD 18, which rose to reach a peak at 2–6 h postpartum (Shynlova et al., 2013b). They also showed increased mRNA and protein levels of cytokines and chemokines that were elevated on GD 18 but peaked at TL and postpartum. Similar results were observed by Edey et al. (2018) *,* who demonstrated an increase in neutrophils and monocytes in the pregnant mouse uterus on GD 18 with a fall at parturition, but the highest levels of cytokine and chemokine mRNA and protein levels were at term parturition on GD 19. Overall, the timing of events, as described by these studies, coincides closely with our demonstrated increases in the abundance of lower uterus chemoattractant (Figure 1B), increased neutrophils in the lower uterus (Figures 3C,D), increased migration of neutrophils (Figure 2), and increased cytokine and chemokine mRNA on GD 18.5 (Figure 5B). Together these actions contribute to promoting the parturient state of the uterus through stimulation of prostaglandins and their receptors, stimulation of more cytokines, and recruitment of more leukocytes. They also contribute to uterine involution that follows delivery of the fetuses and other products of conception (Robertson et al., 1994; Gomez-Lopez et al., 2010; Shynlova et al., 2013a; Shynlova et al., 2013b; Gomez-Lopez et al., 2014; Edey et al., 2018).

Equally intriguing is how IL-1β entices fetal leukocytes to invade the fetal brain. Ly6G + neutrophils were detected in both fetal lung and kidney tissue following maternal administration of sub-labor-inducing LPS doses (Hudalla et al., 2018). While it is likely that neutrophils invaded other fetal tissues, which we did not examine, they would have to overcome the blood-brain barrier to invade the brain. This barrier is compromised in the presence of a severe fetal inflammatory insult (Paton et al., 2017).

IL-1β was administered into the intrauterine space between two adjacent amnionic sacs in this and previous work (Nadeau-Vallee et al., 2015; Nadeau-Vallee et al., 2017). While it is known that cytokines can diffuse between cells (Vitkovic et al., 2000; Oyler-Yaniv et al., 2017), it is unknown if they diffuse from the mother to the fetus. Available data suggest that they propagate from mother to fetus *via* molecular and cellular pathways that include stimulation of their own synthesis in a cell-to-cell fashion (Ishiguro et al., 2016; Leimert et al., 2019a; Leimert et al., 2019b). The same question applies to rytvela, which was administered subcutaneously. Our evidence indicates that it poorly transfers from mother to fetus in the mouse (Nadeau-Vallee et al., 2017). Therefore, rytvela blocks IL-1β action systemically in the dam, which then stops IL-1β from a cell-to-cell stimulation of its own synthesis across the placenta or fetal membranes to the fetus.

The translational implications of this work are significant. Rytvela, our IL-1R allosteric inhibitor, is a formidable therapeutic candidate against PTB and the accompanying long-term physiological consequences on the offspring (Nadeau-Vallee et al., 2017; Coler et al., 2021; Leimert et al., 2021). It selectively inhibits IL-1R signaling down the mitogen-activated protein kinase/p38 pathway that signals for the amplification of the inflammatory cascade that leads to parturition (Nadeau-Vallee et al., 2015). The IL-1R signaling pathways of nuclear factor kappa B is unaffected by rytvela; therefore, it does not interfere with keeping the immunosurveillance intact (Nadeau-Vallee et al., 2015). This is key as infection is responsible for approximately 40% of PTB (Romero et al., 2014).

Having a therapeutic without a diagnostic tool, however, is clinically impractical. A diagnostic would be necessary to inform when treatment with rytvela is required and then used to ascertain when rytvela treatment is successful. Without such a test, there would be no other indicators in cases of subclinical infection or asymptomatic women in the earliest phases of preterm labor to know when treatment is successful. The mLMA measurements of migrated leukocytes on GD 17 in Figure 4 compliment the histological analysis of neutrophil infiltration of the mouse uterus in Figures 3C,D. Indeed, the LMA has the potential to fulfill this diagnostic need. The LMA can be a proxy for assessing silent maternal inflammation and rytvela treatment success before any overt or clinical signs of impending labor are evident.

## Data Availability

The raw data supporting the conclusions of this article will be made available by the authors, without undue reservation.

## Abbreviations

CX, cervix; CCL2, C-C motif chemokine ligand 2; FM, fetal membranes; GD, gestational day; LPS, lipopolysaccharide; LTA, lipoteichoic acid; IL, Interleukin IL-1β and IL-6; IL-1R, interleukin 1 receptor; LMA, leukocyte migration assay; mLMA, mouse leukocyte migration assay; PTB, preterm birth; PL, placenta; PTL, preterm labor; TLR, toll-like receptor (TLR 2 and 4); TL, term labor; TNFα, tumor necrosis factor α; UL, uterus lower; UU, uterus upper.
